# Robotics improves reproducibility of component positioning while producing modest but measurable clinical benefits in total hip arthroplasty: an umbrella review of systematic reviews and meta-analyses

**DOI:** 10.1007/s11701-026-03723-9

**Published:** 2026-07-27

**Authors:** Raju Vaishya, Mohit Kumar Patralekh, Abhishek Vaish, Vikas Khanduja, Filippo Migliorini

**Affiliations:** 1 Department of Orthopaedics, Indraprastha Apollo Hospitals, Sarita Vihar, New Delhi, 110076 India; 2 Vardhman Medical College & Safdarjung Hospital, Ring Road, New Delhi, India; 3 Cambridge Young Adult Hip Service, Department of Trauma & Orthopaedics, Addenbrooke’s - Cambridge University Hospital, , Box 37, Hills Road,, CB2 0QQ Cambridge, UK; 4 Department of Trauma and Reconstructive Surgery, University Hospital of Halle, Martin-Luther University Halle-Wittenberg, Ernst-Grube-Street 40, 06097 Halle (Saale), Germany; 5 Department of Life Sciences, Health, and Health Professions, Link Campus University, Via del Casale di San Pio V, 00165 Rome, Italy; 6 Department of Orthopaedic and Trauma Surgery, Eifelklinik St. Brigida, Kammerbruchstr. 8, 52152 Simmerath, Germany

**Keywords:** Replacement, Complications, Failure, Outcome, Surgery, Biomechanics, Biomaterials

## Abstract

Robotic-assisted total hip arthroplasty (RTHA) promises enhanced implant positioning precision versus conventional THA (CTHA), potentially lowering complications, though evidence from systematic reviews remains inconsistent. The present umbrella review investigates the efficacy of RTHA over CTHA. This umbrella review followed Cochrane guidelines. PubMed, Scopus, and Cochrane Library were accessed. Eleven reviews were included after PRISMA screening. Data on complications, revisions, operative time, leg-length discrepancy (LLD), Forgotten Joint Scores, and alignment (safe zones, cup inclination/anteversion, delta-HCOR/VCOR) were extracted independently. AMSTAR-2 was used to assess methodological quality. The mean difference (MD) and odds ratio (OR) were used, with 95% confidence intervals (CIs). RTHA improved Lewinnek safe-zone placement (OR 7.37, 95% CI 5.51–9.86) and Callanan zone (OR 7.20, 95% CI 5.42–9.55), reduced complications (OR 0.60, 95% CI 0.41–0.87), LLD (MD –1.60 cm, 95% CI –2.30 to -0.90), and Forgotten Joint Scores (MD –6.82, 95% CI –9.81 to –3.83), but increased operative time (MD 15.66 min, 95% CI 10.91–20.41). No differences emerged in revisions (OR 1.08), cup inclination/anteversion, or delta-VCOR. Robust fail-safe N supported findings; funnel asymmetry varied. RTHA enhances precision and reduces short-term complications compared with CTHA, despite longer operative times. Selective use is recommended for high-risk misalignment cases; long-term, cost-effectiveness RCTs are needed.

**Supplementary Information:**

The online version contains supplementary material available at 10.1007/s11701-026-03723-9.

## Introduction

Total hip arthroplasty (THA) remains the definitive treatment for end-stage hip osteoarthritis (OA) and other debilitating conditions, with over 500,000 procedures performed annually in the United States alone and global volumes exceeding two million [1, 2]. While THA achieves excellent long-term pain relief and functional restoration, revision rates remain a substantial clinical burden, with component malposition representing one of the major potentially preventable causes of instability, wear, and early failure [3, 4]. Mal-aligned acetabular cups deviate from the Lewinnek safe zone (40°±10° inclination, 15°±10° anteversion) in up to 50% of conventional cases, leading to early dislocation (2–3% incidence), edge loading, accelerated polyethylene wear, and aseptic loosening [5–7]. Femoral offset and leg-length discrepancies further compound the risk of instability, highlighting the need for precision engineering in component implantation to optimise implant survivorship and minimise reoperation burdens [8].

Conventional THA (CTHA) depends on mechanical guides and freehand techniques, hampered by patient-specific pelvic tilt variations, obesity-related tissue distortion, and surgeon-dependent intraoperative judgments [1, 9]. Robotic-assisted THA (RTHA) addresses these limitations through integrated CT-based 3D preoperative planning, real-time optical navigation, and haptic feedback, enabling sub-millimetre accuracy in cup positioning, version control, and offset restoration, irrespective of surgical volume or learning curve [10–12, 4]. Several Robotic systems have proliferated in the recent past [13–15], yet adoption varies due to high capital costs, prolonged setup times (15–20 min), and learning curves exceeding 20 cases [16–18].

Although several systematic reviews and meta-analyses have evaluated robotic-assisted THA, their conclusions remain inconsistent and are frequently based on overlapping primary studies, heterogeneous robotic platforms, and variable methodological quality [19–24]. To our knowledge, no previous umbrella review has systematically quantified study overlap, critically appraised review quality using AMSTAR-2, and integrated updated re-analyses of the available evidence. The present study was therefore designed to provide a comprehensive higher-level synthesis of the literature, identify areas of consensus and uncertainty, and determine whether the reported advantages of robotic-assisted THA are consistently supported across the existing evidence base. We therefore conducted a PROSPERO-registered (CRD420251242813) umbrella review of systematic reviews/meta-analyses to assess whether RTHA definitively reduces complications, enhances radiographic precision, improves functional outcomes, and alters revision rates compared with CTHA in adult patients.

## Methods

This PROSPERO-registered (CRD420251242813, February 12, 2026) umbrella review of systematic reviews and meta-analyses comparing robotic-assisted (RTHA) versus conventional total hip arthroplasty (CTHA) adhered to the Cochrane Handbook for Systematic Reviews of Interventions and PRISMA guidelines for overviews [25].

### Search strategy

We systematically searched PubMed, Scopus, and Cochrane Library from inception to February 2026 using predefined strings combining MeSH and free-text terms: (“Arthroplasty, Replacement, Hip“[Mesh] OR “total hip arthroplasty” OR THA) AND (“Robotic Surgical Procedures“[Mesh] OR robotic* OR robot-assisted) AND (“Postoperative Complications“[Mesh] OR complication* OR revision*). Filters limited results to meta-analyses/systematic reviews on THA. Reference lists were hand-searched; supplementary file 1 details strategies. No language restrictions applied initially, though non-English texts were excluded post-screening.

## Eligibility criteria (PICOS)

- P (Problem): end-stage hip OA;
- I (Intervention): CTHA;
- C (Comparator): RTHA (any platform);
- O (Outcomes): Complications, revisions, operative time, leg-length discrepancy (LLD), Forgotten Joint Score, radiographic precision (safe-zone placement, cup inclination/anteversion, ΔHCOR/ΔVCOR);
- S (Study design): Systematic reviews with meta-analyses.

## Study selection and data extraction

After deduplication (EndNote X9), two reviewers (RB, SV) independently screened titles/abstracts, then full texts (Kappa agreement: 0.89). Disagreements were resolved by the senior author (RV). Data extracted (duplicate): review characteristics, pooled effect sizes (OR/MD, 95% CI, I²), primary study counts, and outcomes. Primary study data were re-extracted for revised meta-analyses when metrics were inconsistent/incomplete.

## Risk of bias assessment

Included reviews were appraised using AMSTAR-2 [26]; results were visualised as traffic-light plots. Citation overlap was calculated via GROOVE (corrected covered area, CCA) [27]. A CCA of 100% indicates complete overlap, meaning that all reviews include the same primary studies, whereas a CCA of 0% indicates no overlap among reviews. According to previously proposed thresholds, a CCA < 5% indicates slight overlap, 6–10% moderate overlap, 11–15% high overlap, and > 15% very high overlap [27].

## Data synthesis and statistical analysis

Data synthesis and statistical analyses were performed using Review Manager (RevMan, version 5.4.1, The Cochrane Collaboration, Copenhagen, Denmark) and Jamovi (version 2.3.28). Dichotomous outcomes were pooled using odds ratios (ORs) with 95% confidence intervals (CIs), whereas continuous outcomes were pooled using mean differences (MDs) with corresponding 95% CIs. When multiple studies reported the same outcome, pooled estimates were calculated using fixed-effect models for low heterogeneity (I² <50%) and DerSimonian-Laird random-effects models for substantial heterogeneity (I² ≥50%). The pooled effect estimates were derived from re-extracted primary study data after removing duplicate studies identified through overlap analysis. Given the substantial overlap among the included reviews and the variability in reported effect estimates, updated meta-analyses were performed using re-extracted data from the primary studies. This approach was undertaken to provide consistent outcome definitions and comparable pooled estimates across reviews. Duplicate primary studies were identified through cross-referencing and included only once in each analysis. Data synthesis involved Excel^®^ management of extracted outcomes, summarising continuous variables as means ± SD or ranges and categorical variables as counts/percentages, and tabulating review-reported 95% CIs. Primary study data were re-extracted for revised meta-analyses, with overlaps reconciled via GROOVE (CCA = 10.88%) by prioritising complete reports; missing SDs were imputed as (max-min)/4. Heterogeneity was assessed via χ² (*P* < 0.10) and I² (< 50% fixed-effect, otherwise DerSimonian-Laird random-effects models in RevMan 5.4.1/Jamovi 2.3.28), pooling dichotomous outcomes as Mantel-Haenszel ORs and continuous as MDs with 95% CIs. Publication bias was assessed using funnel plots (≥ 10 studies), the Rosenthal fail-safe N, Begg-Mazumdar rank correlation, and Egger regression; significance was set at *P* < 0.05.

## Results

**Fig. 1:**
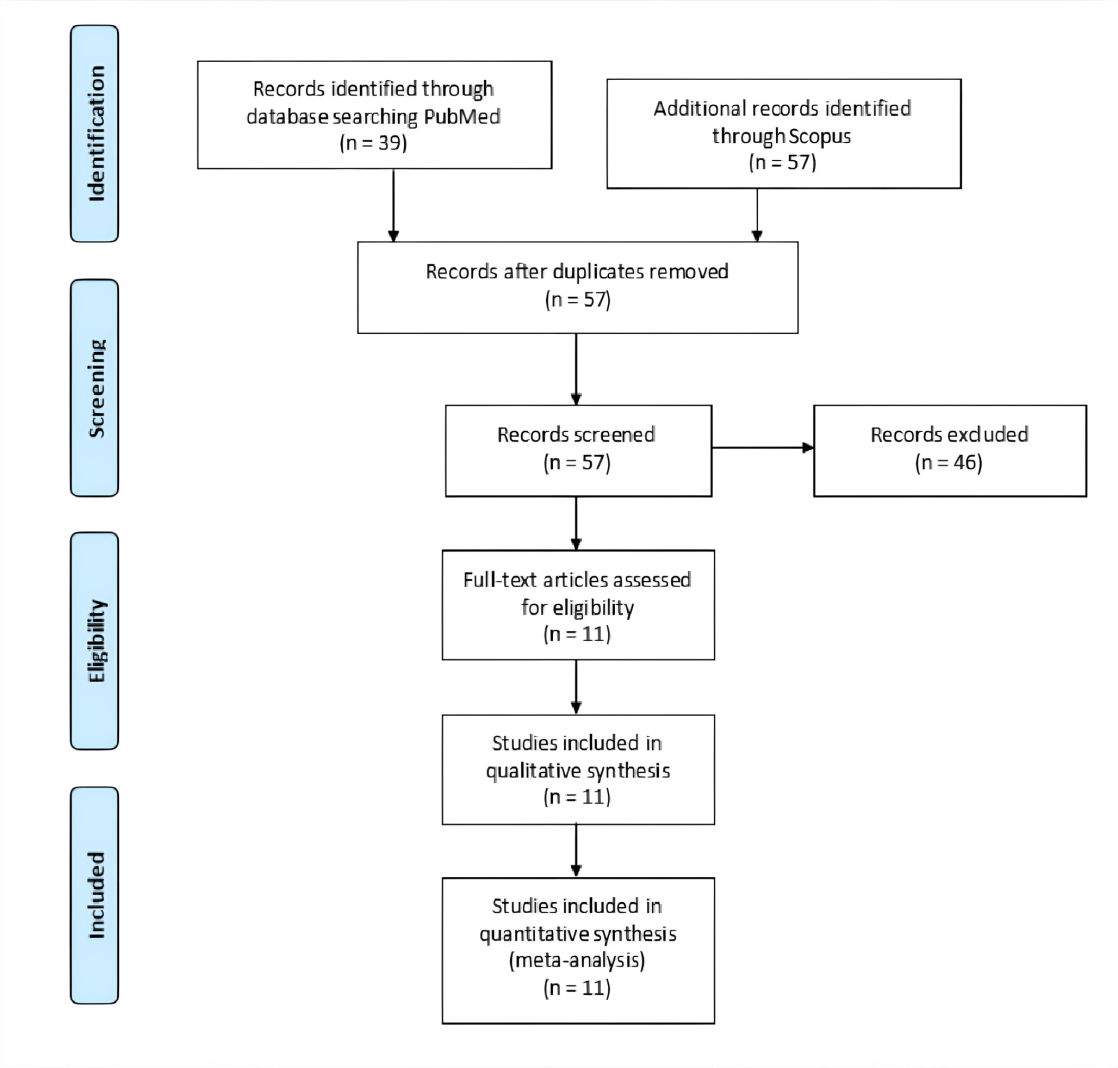
PRISMA flowchart of the study

Database searches (inception to February 2026) across PubMed (*N* = 39), Scopus (*N* = 57), and Cochrane Library (*N* = 0) yielded 96 unique citations after deduplication. Title/abstract screening excluded 85 irrelevant records, leaving 11 systematic reviews/meta-analyses for full-text assessment; all met eligibility criteria (Fig. 1, PRISMA flow diagram). No additional studies were identified through reference screening. GROOVE analysis demonstrated a CCA of 10.88%, indicating a high degree of overlap among the included reviews according to established thresholds.

Table 1 summarises key findings from systematic reviews and meta-analyses comparing RTHA with CTHA. The table highlights differences in implant positioning accuracy, functional outcomes, operative time, complications, revision rates, and limb length discrepancy (LLD) across included studies.

**Table 1:** Key findings of the reviews used in this study

Author	Year	Key Findings
Bensa et al. [28]	2025	Robotic THA showed better implant positioning and fewer complications, but similar functional outcomes; operative time was longer
Llombart-Blanco et al. [29]	2024	Improved cup positioning with MAKO; no meaningful difference in functional scores; complications similar
Chen et al. [20]	2018	Better implant accuracy and fewer intraoperative complications with robotic THA; functional and revision outcomes are similar
Han et al. [24]	2019	Improved cup placement and fewer intraoperative complications with robotic THA; conventional THA had shorter operative time and fewer postoperative complications
Kumar et al. [30]	2023	Robotic THA improved safe zone placement and reduced limb length discrepancy; no difference in complications or long-term outcomes; longer surgery time
Loke et al. [31]	2025	Better implant positioning and slight functional improvement with MAKO; similar complications; operative time marginally longer
Ng et al. [32]	2021	Higher accuracy and slightly better short-term functional outcomes with robotic THA; similar complications; learning curve of 12–35 cases
Ruangsomboon et al. [33]	2024	No difference in complications, revision, or outcomes; robotic THA reduced limb length discrepancy; evidence certainty is low
Tu et al. [34]	2022	Reduced limb length discrepancy and some early functional improvement with robotic THA; longer operative time; similar complications
Wang et al. [35]	2023	Better implant positioning and fewer intraoperative complications with robotic THA; longer surgery time and higher dislocation rates reported
Yin et al. [36]	2026	No major differences in most parameters; slight statistical improvement in HHS and accuracy, but not clinically significant

Revised meta-analyses (Table 2; Supplementary Figs. 1–11) consistently demonstrated RTHA superiority over CTHA across multiple precision and clinical domains. Acetabular cup placement achieved 7-fold higher odds within Lewinnek’s safe zone (OR 7.37, 95% CI 5.51–9.86; *P* < 0.00001; I²=29%, fixed-effects) and Callanan’s zone (OR 7.20, 95% CI 5.42–9.55; *P* < 0.00001; I²=0%), representing the most robust findings with minimal-to-no heterogeneity.

The primary clinical outcome, overall complications, favoured RTHA (OR 0.60, 95% CI 0.41–0.87; *P* < 0.00001; 187/6392 vs. 368/6668 patients), yielding a 40% relative risk reduction despite substantial heterogeneity (I²=60%, random-effects). Patient-centric metrics showed significant improvements: leg-length discrepancy reduced by 1.6 cm (MD –1.60 cm, 95% CI –2.30 to –0.90; I²=89%), Forgotten Joint Scores (FJSs) improved (MD –6.82, 95% CI –9.81 to –3.83; I²=83%), and horizontal centre-of-rotation (HCOR) deviation was minimised (ΔHCOR MD –0.76 mm, 95% CI –0.99 to –0.53; *P* = 0.0001; I²=46%, fixed-effects). RTHA’s sole disadvantage was prolonged operative time (MD 15.66 min, 95% CI 10.91–20.41; *P* < 0.00001; I²=97%), likely attributable to system setup and learning-curve effects.

Neutral findings emerged for revision rates (OR 1.08, 95% CI 0.67–1.76; *P* = 0.75; I²=40%, fixed-effects)—limited by short-term follow-up (< 2 years)—and isolated angular deviations: cup inclination (MD -0.37°, 95% CI –1.45 to 0.71; *P* = 0.50; I²=77%) and anteversion (MD –0.72°, 95% CI –3.10 to 1.66; *P* = 0.55; I²=97%). Vertical centre-of-rotation (VCOR) deviation also showed no difference (ΔVCOR MD -0.38 mm, 95% CI –1.07 to 0.31; *P* = 0.28; I²=87%). These null results for absolute measurements contrast with safe-zone superiority, suggesting that RTHA optimises combined inclination-anteversion targets rather than isolated parameters.

Heterogeneity spanned none (I²=0%, Callanan zone) to considerable (I²=97%, operative time/anteversion), with random-effects models applied when I²≥50% per protocol. Publication bias assessment revealed robust fail-safe N values across outcomes (232–6074; all *P* < 0.001), indicating resistance to unpublished null studies. Funnel plot asymmetry varied predictably: evident in operative time (both tests *P* < 0.0001), LLD (*P* = 0.004–0.019), Forgotten Joint Score (visual), ΔVCOR (visual), and Lewinnek zone (Egger’s *P* = 0.010); symmetric for complications, revisions, cup angles, ΔHCOR, and Callanan zone.

Table 2 provides a comprehensive quantitative summary, accompanied by a methodological legend. AMSTAR-2 quality assessment (Fig. 2), using a traffic-light plot and individual forest plots (Supplementary Figs. 1–11), supplements these findings and confirms methodological rigour despite limitations due to source overlap (Fig. 3).

**Table 2:** Summary of Revised Meta-Analyses: Robotic-Assisted vs. Conventional Total Hip Arthroplasty

Outcome	Effect Measure	RTHA vs. CTHA Result	95% CI	*P*-value	I² (%)	Model	Funnel Symmetry
Lewinnek Safe Zone	OR	7.37	5.51–9.86	< 0.00001	29	Fixed	Egger asymmetric
Callanan Safe Zone	OR	7.20	5.42–9.55	< 0.00001	0	Fixed	Symmetric
Operative Time	MD (min)	15.66	10.91–20.41	< 0.00001	97	Random	Asymmetric
Complications	OR	0.60	0.41–0.87	< 0.00001	60	Random	Symmetric
Leg-Length Discrepancy	MD (cm)	–1.60	–2.30 to -0.90	< 0.00001	89	Random	Asymmetric
Forgotten Joint Score	MD	–6.82	–9.81 to -3.83	< 0.00001	83	Random	Asymmetric
Revisions	OR	1.08	0.67–1.76	0.75	40	Fixed	Symmetric
Cup Inclination	MD (°)	–0.37	–1.45 to 0.71	0.50	77	Random	Symmetric
Cup Anteversion	MD (°)	–0.72	–3.10 to 1.66	0.55	97	Random	Symmetric
ΔHCOR	MD (mm)	–0.76	–0.99 to -0.53	0.0001	46	Fixed	Symmetric
ΔVCOR	MD (mm)	–0.38	–1.07 to 0.31	0.28	87	Random	Asymmetric
Overlap (GROOVE)	CCA	10.88	–	–	–	–	–

(OR = odds ratio (> 1 favours RTHA for binary outcomes); MD = mean difference (< 0 favours RTHA for continuous outcomes); CI = confidence interval; I² = heterogeneity (%); Fixed-effect model (I²<50%); Random-effects model (I²≥50%). Funnel symmetry was assessed via Begg-Mazumdar rank correlation, Egger’s regression, Rosenthal fail-safe N, and visual inspection. “Egger asymmetric” indicates only a significant regression test. Overlap via GROOVE corrected covered area (CCA)

**Fig. 2:**
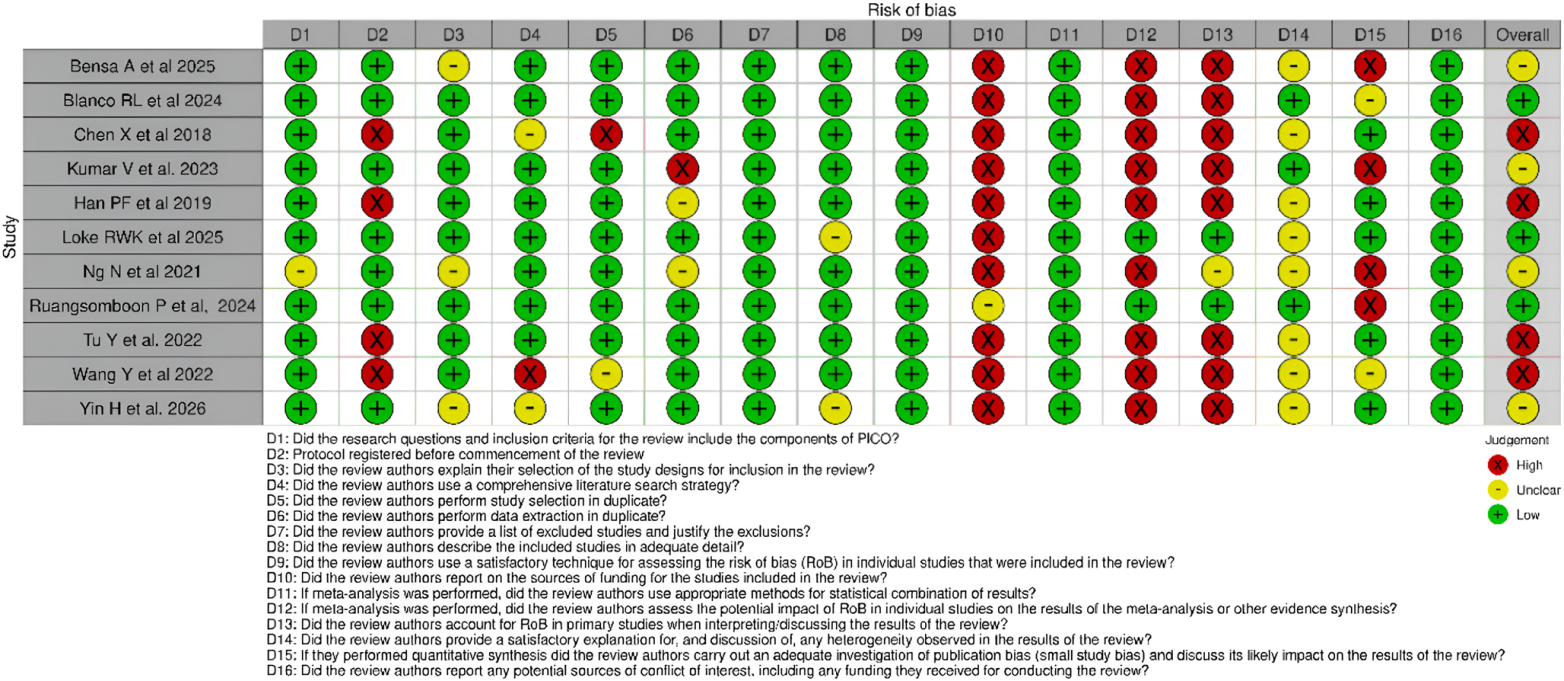
Study quality/risk of bias assessment using AMSTAR 2 traffic light plot

**Fig. 3:**
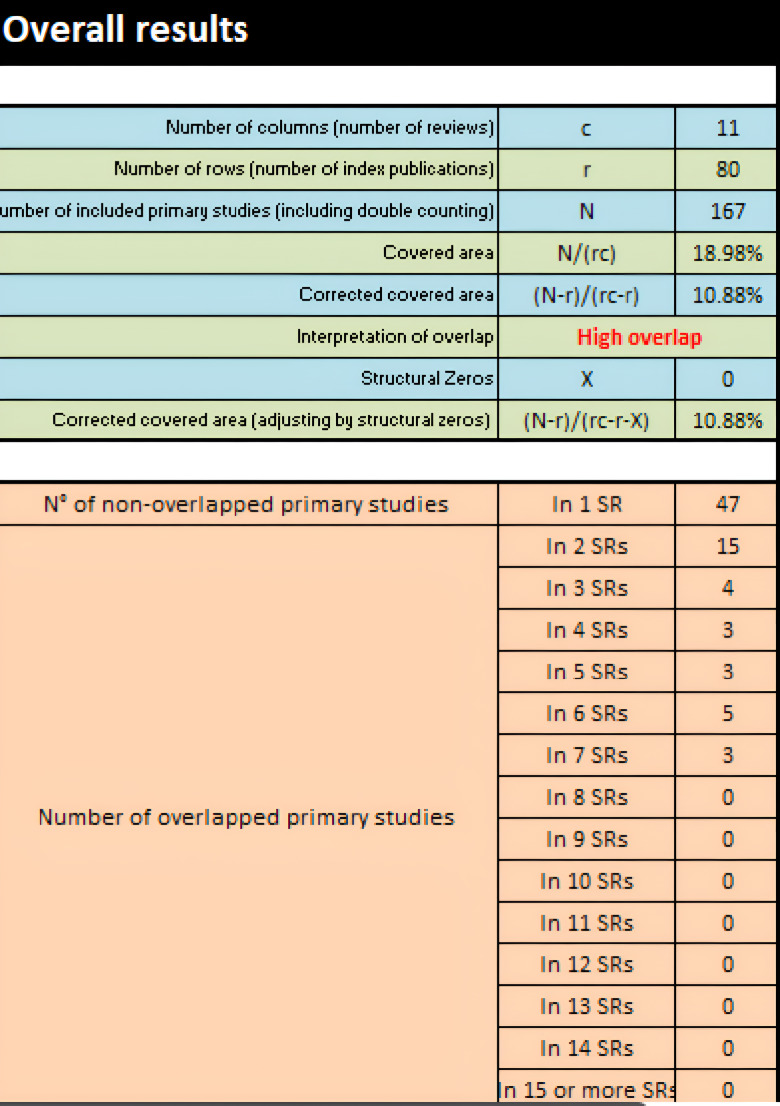
Overlap analysis revealing a relatively high overlap of primary studies

## Discussion

This umbrella review synthesises 11 systematic reviews/meta-analyses, confirming the superiority of RTHA over CTHA in radiographic precision and short-term clinical outcomes. Although several outcomes reached statistical significance, their clinical relevance should be interpreted carefully. Improvements in radiographic parameters, safe-zone achievement, and restoration of hip biomechanics do not necessarily translate into perceptible benefits for patients, particularly in the short term. Similarly, statistically significant differences in patient-reported outcomes may not always exceed established thresholds for minimal clinically important differences [37, 38]. Therefore, the observed advantages of RTHA should be interpreted within the broader context of patient-centred outcomes and long-term implant performance. Seven of nine key metrics significantly favoured RTHA: 7-fold higher safe-zone placement (Lewinnek OR 7.37, 95% CI 5.51–9.86; Callanan OR 7.20) [39], reduced complications (OR 0.60) [20], leg-length discrepancy (MD -1.60 cm), FJS (MD -6.82), and ΔHCOR (MD -0.76 mm). These align with RTHA’s CT-integrated planning, optical tracking, and haptic feedback, achieving > 90% safe-zone rates versus 35–50% in CTHA [16]. Nevertheless, the clinical significance of achieving the traditional Lewinnek safe zone remains a matter of ongoing debate [40, 41]. Contemporary evidence suggests that implant stability and functional outcomes are influenced by a complex interplay of factors, including spinopelvic mobility, patient-specific anatomy, soft-tissue balance, and functional component orientation [42, 43]. Therefore, improved safe-zone attainment should primarily be interpreted as a marker of enhanced surgical precision rather than a direct surrogate for clinical success. Optimal positioning mitigates edge loading, impingement, and wear mechanisms that underlie 70% of early THA failures [5]. Overall complications occurred in 2.9% of robotic cases compared with 5.5% of conventional procedures, corresponding to a relative risk reduction of approximately 40%. The 40% complication reduction (dislocation, fracture, infection) mirrors prior meta-analyses reporting ORs of 0.4–0.7 [20, 39], though our higher safe-zone effects stem from primary re-analysis, minimising aggregation bias.

Heterogeneity (I² 0–97%) reflects surgeon experience (learning curve > 20 cases) [21], platform variability (MAKO in 70% of studies vs. ROSA/OMNI), patient factors (BMI, dysplasia), and outcome definitions. For several outcomes, including operative time, cup anteversion, leg-length discrepancy, and Forgotten Joint Score, heterogeneity was considerable (I² >80%). Therefore, these pooled estimates should be interpreted cautiously, as they may reflect differences in robotic platforms, patient characteristics, surgical approaches, and outcome assessment methods rather than a uniform treatment effect [44]. Robust fail-safe N (232–6074) across analyses resists the nullification of unpublished studies, enhancing confidence despite occasional funnel asymmetry (e.g., operative time, LLD). Longer RTHA times (MD 15.66 min) primarily arise from setup/registration (10–15 min) [45], with efficiency gains post-proficiency (plateau at ~ 15 cases) [46]. Cost-benefit analyses project a break-even at 50–100 cases/year in high-volume centres [47], given reduced revisions (~$30,000 in savings/event) [48]. Null findings for revisions (OR 1.08), isolated cup angles, and ΔVCOR highlight short-term data limitations (median follow-up < 2 years); precision benefits are likely to accrue over the long term via reduced wear/loosening [11, 6, 49, 50]. A 10-year case series reported 93 HHS, 85% FJS-12 satisfaction, and low revision rates with RTHA [51], but RCTs with 5 + year endpoints are scarce. Navigation trials show similar trends (OR ~ 0.5 complications), but RTHA’s haptics confer 2–3° superior accuracy [19]. Head-to-head RCTs comparing robotics vs. navigation are thus warranted. AMSTAR-2 critiques (traffic-light plot) identified moderate risks in 7/11 reviews (e.g., undisclosed funding), though our reanalyses mitigate risk propagation. High overlap (CCA 10.88%) amplifies influential primaries, inflating precision ORs. Substantial overlap reduces the independence of the available evidence, as the same primary studies contribute repeatedly across multiple evidence syntheses. Consequently, the apparent consistency of findings across reviews should be interpreted with caution, as it may partly reflect repeated inclusion of influential studies rather than entirely independent confirmation of the observed effects. Industry sponsorship (80% of robotics literature) risks bias [52, 53], though effect sizes exceed those of prior industry-unadjusted meta-analyses [22]. This consideration is particularly relevant in robotic arthroplasty, where most published studies evaluate proprietary platforms developed by a limited number of manufacturers [54]. Industry involvement may influence study design, comparator selection, outcome reporting, and publication patterns, potentially favouring positive findings [53, 52]. Although industry-supported research has substantially contributed to technological innovation and evidence generation, independent studies and registry-based analyses remain essential to confirm the reproducibility and generalisability of reported benefits across different healthcare settings. RTHA adoption suits high-risk cohorts (dysplasia, neuromuscular disease, obesity) in which malposition exceeds 60%, potentially halving dislocation rates (0.7% vs. 2%) [19, 55]. Selective use optimises resource allocation amid rising demand for THA (projected to increase by 572% in the US by 2030) [56]. Functional gains (FJS, LLD) enhance satisfaction and address litigation drivers [57, 58]. RTHA, including in revision settings, is increasingly supported by evidence that it enhances precision in acetabular reconstruction and implant positioning through improved preoperative assessment of bone defects and intraoperative guidance. Early clinical and radiological outcomes are satisfactory, with additional system-level benefits, including reduced LOS without affecting discharge disposition, suggesting potential cost advantages in high-demand settings [59]. The literature also highlights improved component alignment, preservation of bone stock, early functional recovery, and a relatively short learning curve [4, 17]. However, despite these promising findings, adoption is limited by high costs, infrastructure requirements, and technical challenges in complex anatomies, emphasising the need for long-term outcomes and cost-effectiveness analyses to better define its role in routine clinical practice [54, 47, 60].

This study has several limitations. Different robotic platforms were pooled together despite relevant differences in hardware, software, registration techniques, and surgeon interaction, potentially masking platform-specific effects. Similarly, the type of implants and surgical access, which were often poorly described, were highly heterogeneous. High primary study overlap (CCA 10.88%) risks amplifying biases from influential trials and inflating effect sizes. Most data derive from short-term follow-ups (< 2 years), underpowering revision/complication endpoints; industry-funded primaries (common in robotics) may introduce sponsorship bias, which is not mitigated by AMSTAR-2 critiques. Search limitations (no Embase, English-only) and SD imputations introduce uncertainty, while sources of heterogeneity (e.g., patient demographics, surgical volume) remain unexplored; GRADE assessments are absent, limiting certainty grading. The substantial heterogeneity observed for several outcomes may limit the precision and generalisability of the pooled estimates. Consequently, findings for highly heterogeneous outcomes should be interpreted as indicative rather than definitive. Long-term (> 5-year) multicenter RCTs should stratify by surgeon volume/platforms and incorporate cost-utility (QALYs). Real-world registries will validate generalizability beyond elective cohorts [34]. Head-to-head vs. navigation, AI augmentation, and patient-specific implants merit exploration [46].

## Conclusions

Robotic-assisted THA demonstrated superior implant positioning accuracy and reduced complication rates compared with conventional THA. Improvements in selected patient-reported and radiographic outcomes were observed, although evidence for long-term functional superiority and implant survivorship remains limited. Further high-quality studies with extended follow-up are required to determine the clinical and economic value of widespread adoption.

## Supplementary Information

Below is the link to the electronic supplementary material.

Supplementary Material 1

## Data Availability

The datasets generated during and/or analysed during the current study are available throughout the manuscript.

## Declarations

### Registration and protocol

This umbrella review was registered in PROSPERO under the registration number CRD420251242813.

### Use of AI

Language editing was performed using Grammarly (Superhuman Platform Inc., San Francisco, CA, USA).
