# Supplementary material for: Robotics improves reproducibility of component positioning while producing modest but measurable clinical benefits in total hip arthroplasty: an umbrella review of systematic reviews and meta-analyses
Source: J Robot Surg. 2026 Jul 27;20(1):757. doi: 10.1007/s11701-026-03723-9 (PMC13402262; doi:10.1007/s11701-026-03723-9)
Supplement: Supplementary file 1 — Supplementary Material 1 [file 11701_2026_3723_MOESM1_ESM.docx]

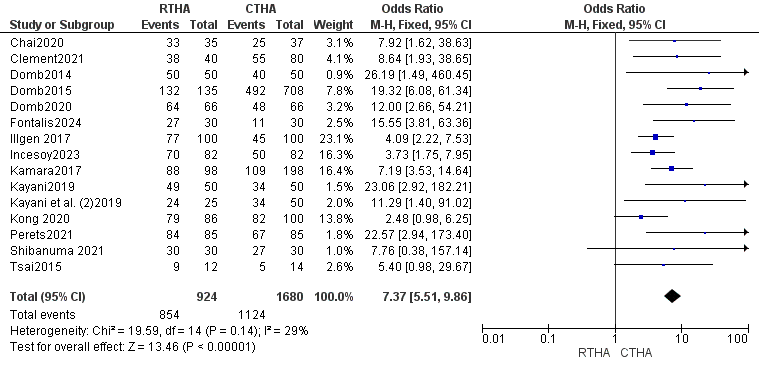


**Supplementary Figure 1A:** Forest plot on implant positioning within Lewinnek safe zone demonstrating significantly better positioning in the RTHA group (OR: 7.37; 95% CI: 5.51, 9.86; P< 0.00001)


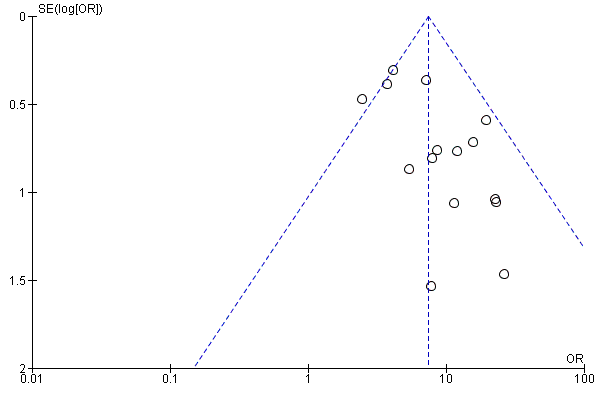

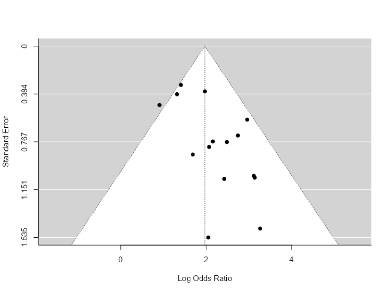


**Supplementary Figure 1B:** Funnel plot on implant positioning within Lewinnek safe zone; Begg and Mazumdar's rank correlation test could not detect asymmetry (p = 0.4351), but Egger’s regression test indicated significant funnel plot asymmetry (p = 0.0101)


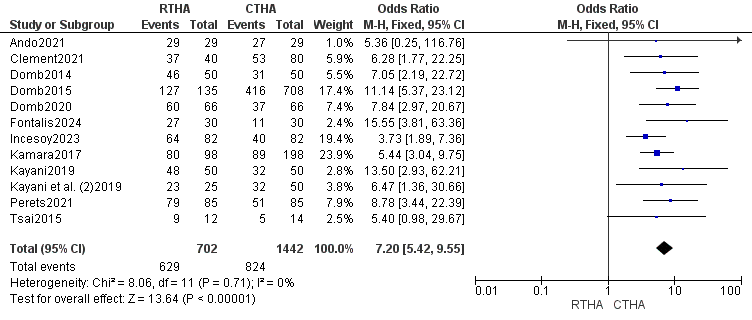


**Supplementary Figure 2A:** Forest plot on implant positioning within Callanan safe zone demonstrating significantly better positioning in the RTHA group (OR: 7.20; 95% CI: 5.42, 9.55; P< 0.00001).


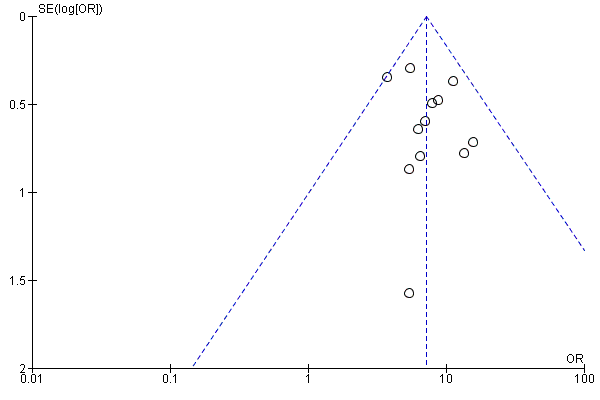

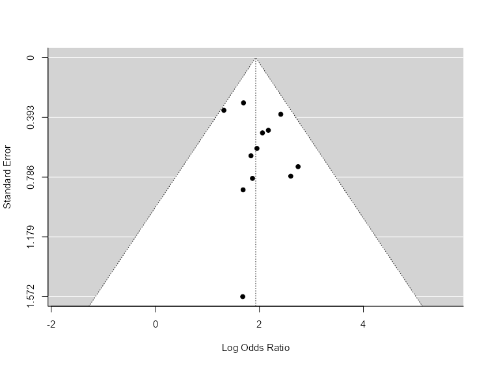


**Supplementary 2B:** Funnel plot on implant positioning within Callanan safe zone; neither the rank correlation nor the regression test indicated any funnel plot asymmetry (p = 0.7373 and p = 0.4005, respectively).


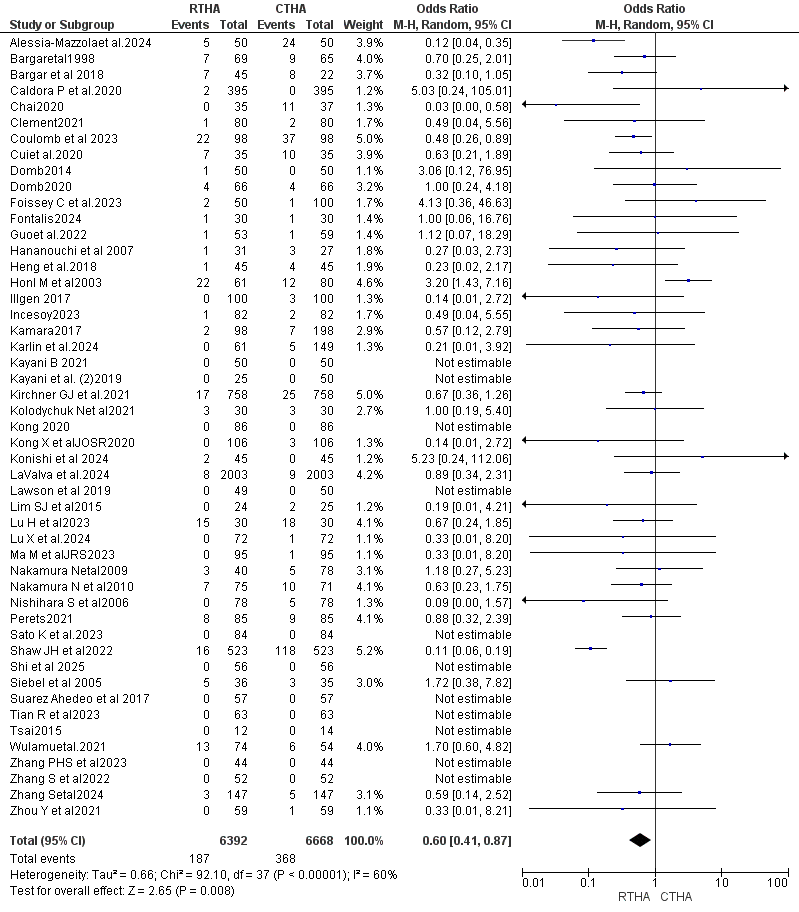


**Supplementary Figure 3A:** Forest plot on operative time demonstrating significantly higher operative time for the RTHA group with a mean difference (MD) of 15.66 minutes and a 95% CI of 10.91 to 20.41, p<0.00001.


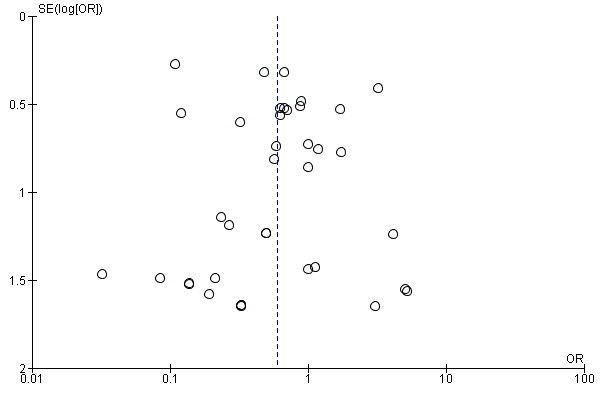

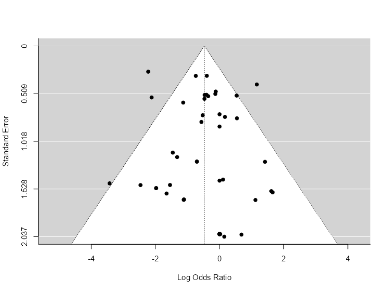


**Supplementary Figure 3B:** Funnel plot on operative time; both the rank correlation and the regression test indicated potential funnel plot asymmetry (p = 0.0002 and p < 0.0001, respectively)


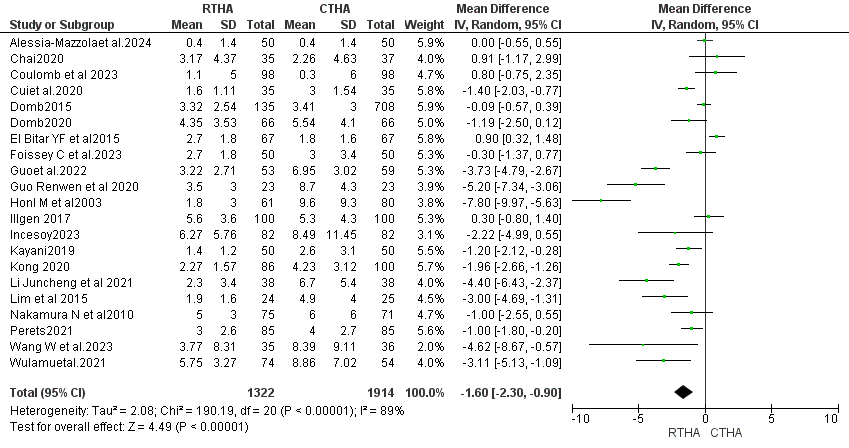


**Supplementary Figure 4A:** Forest plot demonstrating significantly lower complication rate in the RTHA group (187/6392) as compared to the CTHA group (368/6668) (OR: 0.60; 95% CI: 0.41, 0.87; P< 0.00001).

**
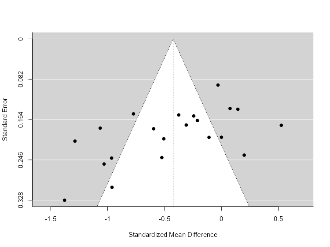
**
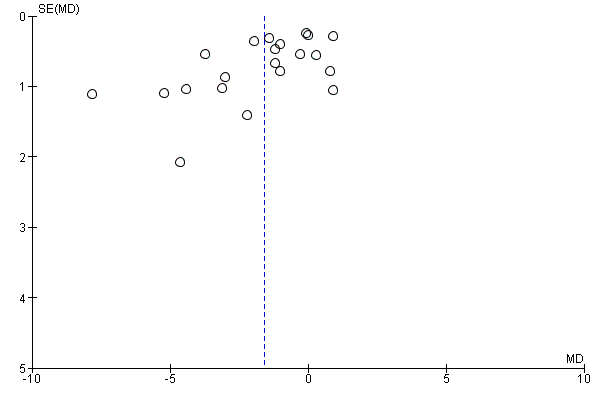


**Supplementary Figure 4B:** Funnel plot on complication rate; neither the rank correlation nor the regression test indicated any funnel plot asymmetry (p = 0.6619 and p = 0.7403, respectively).


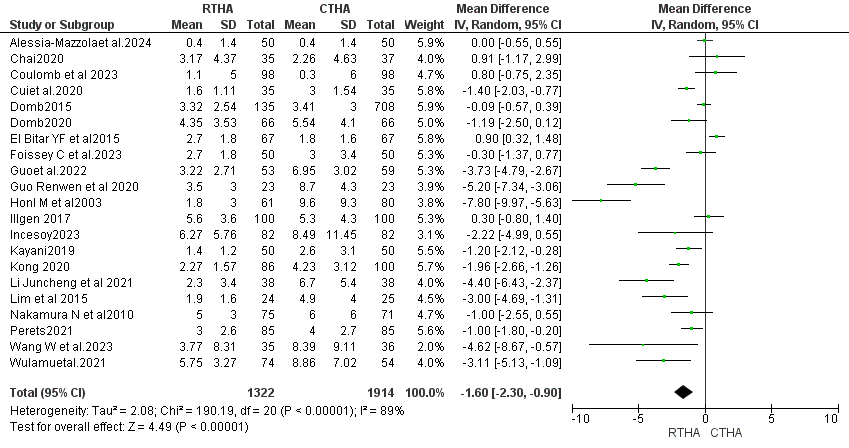


**Supplementary Figure 5A:** Forest plot demonstrating significantly lower LLD for the RTHA group, with a mean difference (MD) of 1.60 cm and a 95% CI of 2.30 to 0.90, p<0.00001.

**
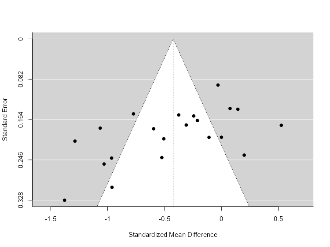
**
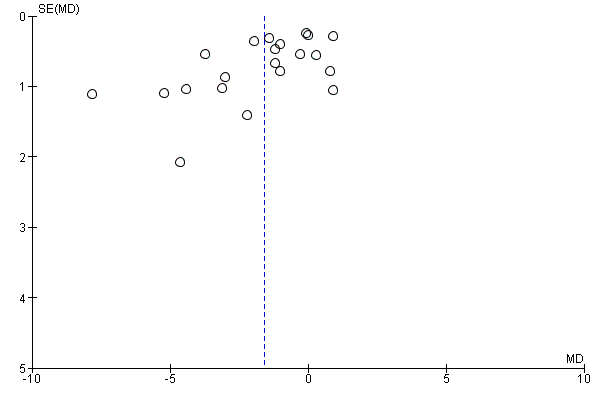


**Supplementary Figure 5B:** Funnel plot on LLD; both the rank correlation and the regression test indicated potential funnel plot asymmetry (p = 0.0187 and p = 0.0040, respectively


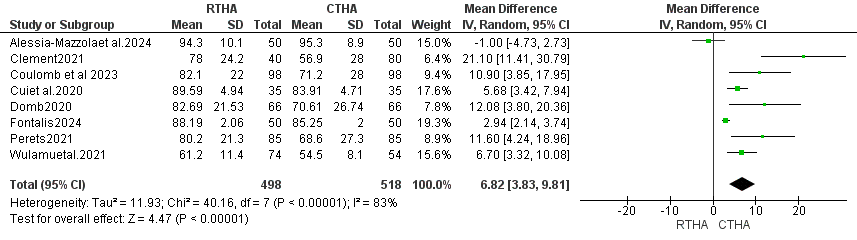


**Supplementary Figure 6A:** Forest plot on forgotten joint scores revealed significantly lower values for the RTHA group with a mean difference (MD) of 6.82 and a 95% CI of 3.83 to 9.81, p<0.00001


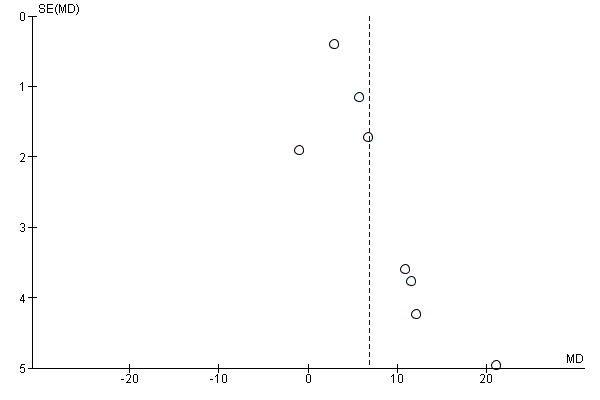


**Supplementary Figure 6B:** Funnel plot on forgotten joint scores with asymmetry on visual inspection.


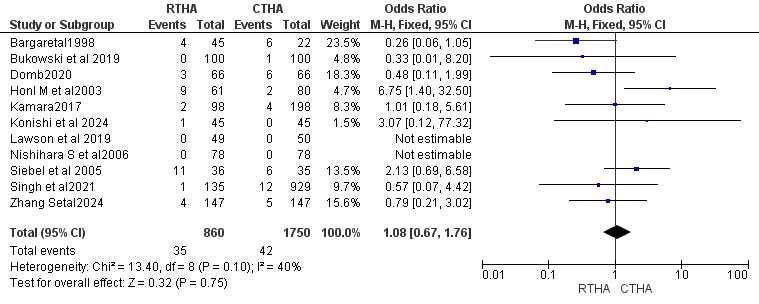


**Supplementary Figure 7A:** Forest plot demonstrating no significant difference in revision rates between the RTHA and CTHA groups (OR: 1.08; 95% CI: 0.67, 1.76; P = 0.75)


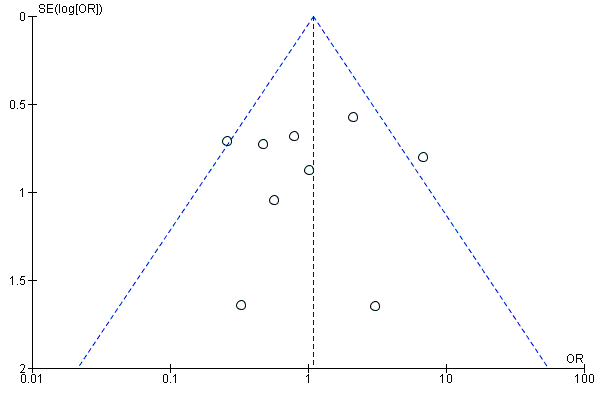
**
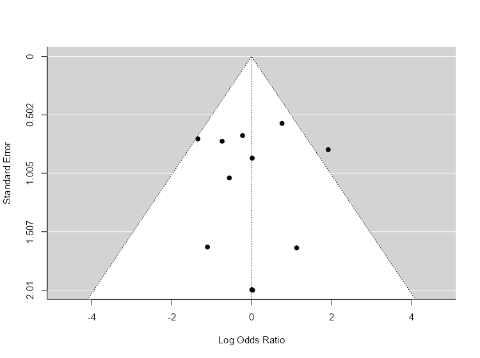
**

**Supplementary Figure 7B:** Funnel plot on revision rates; neither the rank correlation nor the regression test indicated any funnel plot asymmetry (p = 0.7612 and p = 0.9483, respectively).


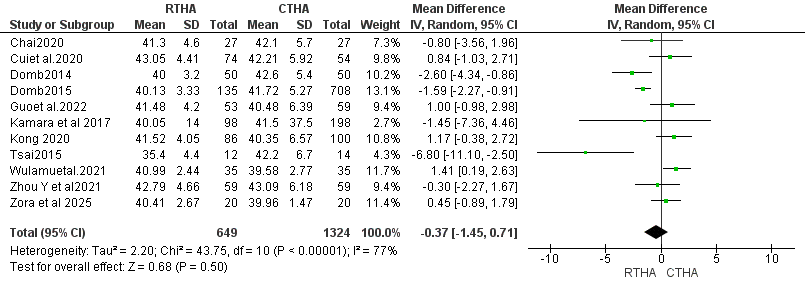


**Supplementary Figure 8A:** Forest plot demonstrating no significant difference in cup inclination with a mean difference (MD) of -0.37 and a 95% CI of -1.45 to 0.71, p = 0.50.


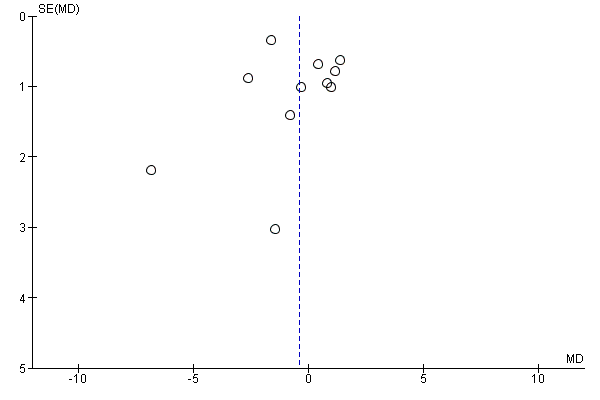

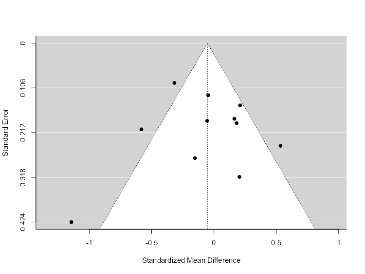


**Supplementary Figure 8B:** Funnel plot on cup inclination; neither the rank correlation nor the regression test indicated any funnel plot asymmetry (p = 1.0000 and p = 0.4368, respectively).


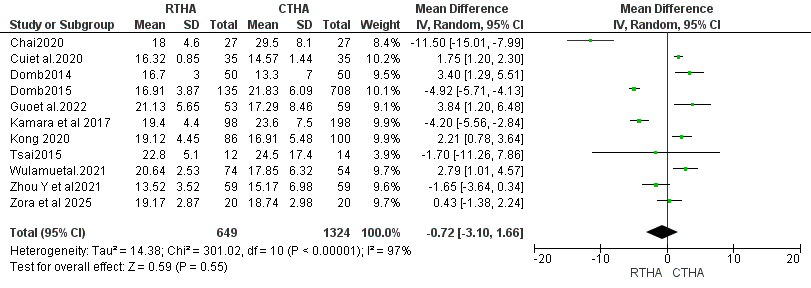


**Supplementary Figure 9A:** Forest plot demonstrating no significant difference in cup anteversion with an MD of -0.72 and a 95% CI of -3.10 to 1.66, p= 0.55.


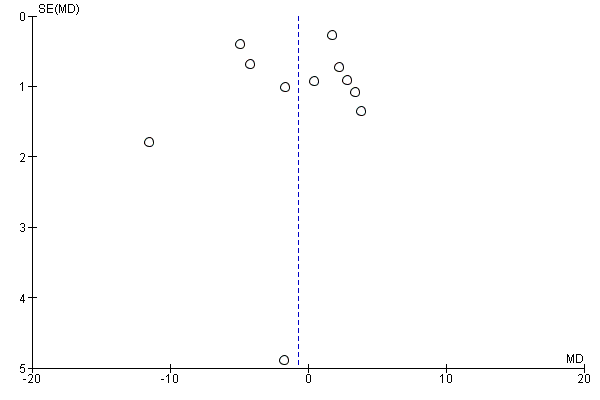

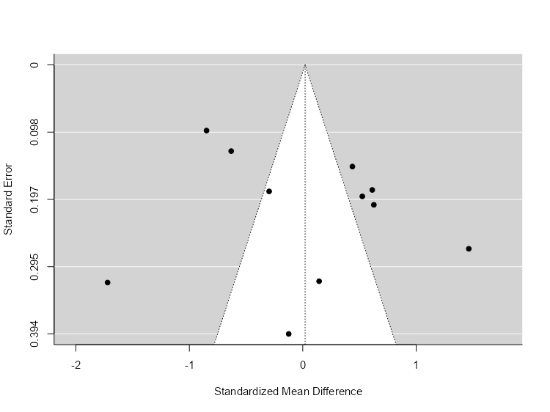


**Supplementary Figure 9B:** Funnel plot on cup anteversion; neither the rank correlation nor the regression test indicated any funnel plot asymmetry (p = 0.7612 and p = 0.9733, respectively).


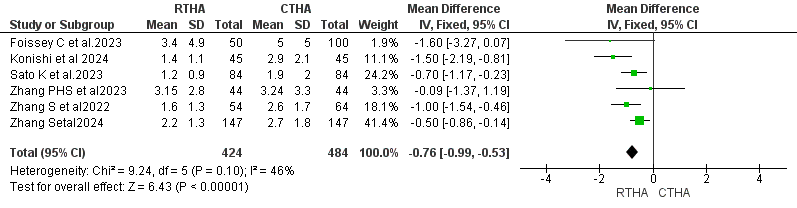


**Supplementary Figure 10A:** Forest plot demonstrating a significant difference in change in horizontal centre of rotation in favour of the RTHA group with an MD of -0.76 and a 95% CI of -0.99 to -0.53, p < 0.0001.


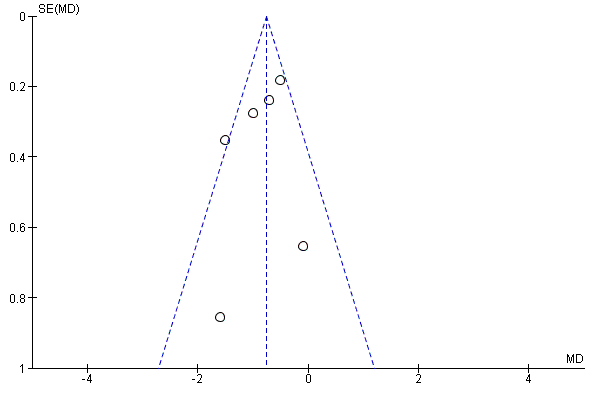


**Supplementary Figure 10B:** Funnel plot on change in HCOR, without any asymmetry on visual inspection.


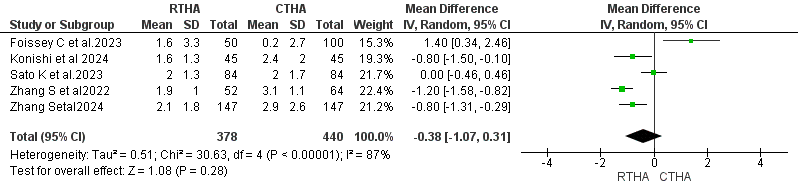


**Supplementary Figure 11A:** Forest plot demonstrating no significant difference in change in the vertical centre of rotation between the RTHA and CTHA groups with an MD of -0.38 and a 95% CI of -1.07 to 0.31 (p = 0.28).


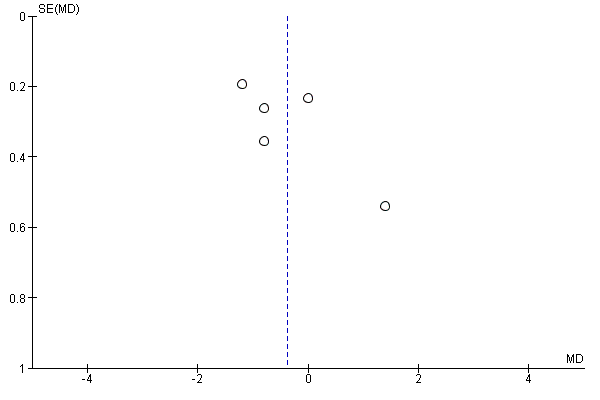


**Supplementary Figure 11B:** Funnel plot on delta VCOR with asymmetry on visual inspection.
